# Comparison of rice and whey protein osolate digestion rate and amino acid absorption

**DOI:** 10.1186/1550-2783-10-S1-P12

**Published:** 2013-12-06

**Authors:** Ralf Jäger, Joshua E Dudeck, Jordan M Joy, Ryan P Lowery, Sean A McCleary, Stephanie MC Wilson, Douglas S Kalman, Jacob M Wilson, Martin Purpura

**Affiliations:** 1 Increnovo LLC, 2138 E Lafayette Pl, Milwaukee, WI, USA; 2 Department of Health Sciences and Human Performance, The University of Tampa, Tampa, FL, USA; 3 Department of Nutrition, IMG Performance Institute, IMG Academy, Bradenton, FL, USA; 4 Department of Nutrition and Endocrinology, Miami Research Associates, Miami, FL, USA

## Background

Athletes have a choice of different animal (e.g. whey, casein, egg, beef, fish) or plant protein (e.g. soy, rice, pea, hemp) sources, which differ in numerous ways such as the presence of allergens (lactose, soy), cholesterol, saturated fats, digestion rate (fast, intermediate, or slow absorption of amino acids), or the relative amount of individual amino acids. While digestibility of rice protein isolate (RPI) in rats has been shown to be inferior to animal protein (87% vs. 97% for casein), administration of 48 grams of RPI following resistance exercise decreased fat-mass and increased lean body mass, skeletal muscle hypertrophy, power and strength comparable to whey protein isolate (WPI). This study sought to investigate the amino acid rate of appearance in the blood of 48 grams of RPI compared to 48 grams of WPI.

## Methods

After a 12 hour overnight fast, 10 subjects (22.2 ± 4.2 years of age, bodyweight of 77.4 ± 0.6 kg, and height of 176.8 cm ± 8.6 cm) were randomly assigned to receive either 48 grams of RPI (Growing Naturals Rice Protein Isolate (Chocolate Power) made with Oryzatein® rice protein, Axiom Foods, Oro Valley, AZ) or WPI (Nutra Bio Whey Protein Isolate (Dutch Chocolate), Middlesex, NJ) in a double-blind, crossover design, separated by a washout phase of 7 days. Blood draws were taken immediately prior to, and at 1, 2, 3, and 4 hours following consumption of WPI or RPI.

## Results

WPI and RPI showed a significant difference for Tmax for essential amino acids (EAA: RPI 87 ± 7 min, WPI 67 ± 4 min, p=0.03), non-essential amino acids (NEA: RPI 97 ± 4 min, WPI 71 ± 5 min, p<0.001), and total amino acids (TA: RPI 93 ± 4 min, WPI 69 ± 3 min, p<0.001), however no significant differences were detected for AUC (EAA: RPI 649.5 ± 140.9 nmol/ml, WPI 754.2 ± 170.0 nmol/ml, p=0.64; NEA: RPI 592.7 ± 118.2 nmol/ml, WPI 592.7 ± 121.2 nmol/ml, p=0.98; TA: RPI 615.9 ± 88.6 nmol/ml, WPI 661.1 ± 98.7 nmol/ml, p=0.74), and Cmax (EAA: RPI 176.1 ± 37.5 nmol/ml, WPI 229.5 ± 51.2 nmol/ml, p=0.41; NEA: RPI 160.0 ± 31.1 nmol/ml, WPI 178.4 ± 34.0 nmol/ml, p=0.69; TA: RPI 166.6 ± 23.4 nmol/ml, WPI 199.3 ± 28.8 nmol/ml, p=0.38). On an individual amino acid basis, WPI and RPI showed bioequivalency (0.80-1.25 of the geometic mean ratio (GMR)) for AUC and Cmax for all amino acids with the exception of cystine, isoleucine, leucine, lysine, and threonine, in which WPI performed significantly better. Tmax differed between WPI and RPI for histadine, phenelyalanine, threonine, asparagine, glutamic acid, glycine, ornithine, proline, and serine.

## Conclusion

These findings suggest that RPI, compared to WPI (fast) and casein (slow), is an intermediate digesting protein. While RPI showed a 6.8% lower total amino acid appearance in the blood based on AUC, the difference was not statistically significant. Future research should investigate the digestion kinetics of RPI for longer periods of time, potentially reducing the observed difference in total amino acid appearance in the blood due to the difference in digestion rates of WPI (fast) and RPI (intermediate). In addition, the potential nutritional effects of the significant differences in absorption of some of the individual amino acids, based on different amino acid content and absorption kinetics of the protein sources, warrants further research.

